# Is He Being Bad? Social and Language Brain Networks during Social Judgment in Children with Autism

**DOI:** 10.1371/journal.pone.0047241

**Published:** 2012-10-17

**Authors:** Elizabeth J. Carter, Diane L. Williams, Nancy J. Minshew, Jill F. Lehman

**Affiliations:** 1 Robotics Institute, Carnegie Mellon University, Pittsburgh, Pennsylvania, United States of America; 2 Department of Psychology, Carnegie Mellon University, Pittsburgh, Pennsylvania, United States of America; 3 Department of Speech-Language Pathology, Duquesne University, Pittsburgh, Pennsylvania, United States of America; 4 Departments of Neurology and Psychiatry, University of Pittsburgh School of Medicine, Pittsburgh, Pennsylvania, United States of America; 5 Department of Computer Science, Carnegie Mellon University, Pittsburgh, Pennsylvania, United States of America; Ecole Normale Supérieure, France

## Abstract

Individuals with autism often violate social rules and have lower accuracy in identifying and explaining inappropriate social behavior. Twelve children with autism (AD) and thirteen children with typical development (TD) participated in this fMRI study of the neurofunctional basis of social judgment. Participants indicated in which of two pictures a boy was being bad (Social condition) or which of two pictures was outdoors (Physical condition). In the within-group Social–Physical comparison, TD children used components of mentalizing and language networks [bilateral inferior frontal gyrus (IFG), bilateral medial prefrontal cortex (mPFC), and bilateral posterior superior temporal sulcus (pSTS)], whereas AD children used a network that was primarily right IFG and bilateral pSTS, suggesting reduced use of social and language networks during this social judgment task. A direct group comparison on the Social–Physical contrast showed that the TD group had greater mPFC, bilateral IFG, and left superior temporal pole activity than the AD group. No regions were more active in the AD group than in the group with TD in this comparison. Both groups successfully performed the task, which required minimal language. The groups also performed similarly on eyetracking measures, indicating that the activation results probably reflect the use of a more basic strategy by the autism group rather than performance disparities. Even though language was unnecessary, the children with TD recruited language areas during the social task, suggesting automatic encoding of their knowledge into language; however, this was not the case for the children with autism. These findings support behavioral research indicating that, whereas children with autism may recognize socially inappropriate behavior, they have difficulty using spoken language to explain why it is inappropriate. The fMRI results indicate that AD children may not automatically use language to encode their social understanding, making expression and generalization of this knowledge more difficult.

## Introduction

Autism has three key characteristics: impairments in social interactions, reduced communication skills, and restricted interests and repetitive behaviors [1]. Of these, the social abnormalities are unique to autism [1], [2]. In Kanner's original description [2], he detailed a number of socially inappropriate behaviors, including playing alongside rather than with others, a preference for aloneness and an indifference to others, neither asking nor answering questions, and pushing people away. Individuals with autism may show increased levels of interpersonal aggression (e.g., [3], [4]); deficits in emotional regulation [5], including high levels of tantrums [6], [7]; as well as reduced positive interaction behaviors [8].

In addition to having a more difficult time acting appropriately in interpersonal contexts, children with autism perform poorly when they have to judge the behaviors of others. A number of behavioral studies have investigated various aspects of social judgments in children with autism. Using videotaped vignettes with varying numbers of social cues, Pierce and colleagues [9] asked children whether characters were mean or nice and whether they acted appropriately. The responses of the children with autism were compared to two groups matched on verbal mental age: younger children with typical development and age-matched children with mental retardation. The children with autism performed worse on the social questions, particularly as the number of cues that required attention increased, but performed as well as the other groups of children on factual questions. Similarly, in another study, high-functioning children with autism or Asperger syndrome performed more poorly than children with typical development on identifying faux pas in auditorily-presented stories, despite equivalent performance on false belief tasks [10]. Loveland and colleagues [11] created a task for discriminating between appropriate and inappropriate behavior that also modulated whether the videos used verbal or nonverbal cues. The children and adolescents with autism had more difficulty correctly identifying inappropriate verbal behaviors than the children with typical development. Moreover, they gave unusual reasoning for their selections. In another study, older children with autism were asked to make moral judgments about deliberate or accidental actions against people or property [12]. The children with autism performed similarly to children with typical development and children with learning difficulties when determining that motive and consequences were important. However, the children with autism were unable to verbally justify their reasoning in the majority of cases, and the appropriateness of justifications correlated with verbal mental age in the children with autism but not the children with typical development [13]. In a recent study, when 9- to 13-year-old children with autism rated the social appropriateness of a range of stories, they rated more normal behaviors as strange than did the children with typical development, even though the two groups performed equivalently on inappropriate behaviors [14]. Additionally, the children with autism provided more bizarre explanations and refusals to answer (e.g., “I don't know” or silence) than the comparison group. In summary, children with autism often have difficulty making social judgments accurately, and even when they are successful, they often cannot verbally justify their answers.

Multiple mechanisms have been proposed to underlie this unusual performance. One suggestion is that it is a function of underlying deficits in attention, given poorer performance with increased numbers of cues [9]. Some research [11], [14] has implicated abnormal language processing as a possible explanation for poor performance because of the reduced ability to verbally explain judgments. The correlation of verbal mental age in children with autism with the quality of their justifications [12] further suggests that impaired language skills could be an important factor in the difficulty children with autism have in making social judgments about the behavior of others. If the difference is related to language skills, the assumption would be that the reduced overall accuracy in certain social judgment tasks reflects the necessity of language use in those tasks even when measures other than verbal justification are used.

However, theory of mind could also be an important factor in making social judgments. Theory of mind, or mentalizing, is the ability to recognize that other individuals have thoughts, beliefs, desires, and other mental states that are distinct from one's own [15]. To interpret the behavior of others in context, it is often important to understand their thoughts and motivation. Behavioral studies have long suggested that there is reduced mentalizing ability in autism (e.g., [16], [17]), which would then affect their ability to make social judgments. Additionally, recent research has demonstrated mentalizing impairments in young children with autism even when language is not required for the theory-of-mind task [18], suggesting independence of the two processes.

Theory of mind is believed to rely upon the medial prefrontal cortex (mPFC), posterior superior temporal sulcus (pSTS), temporoparietal junction (TPJ), and temporal poles (for reviews, see [19], [20]). Many neuroimaging studies have shown reduced activation in key theory-of-mind brain regions in individuals with autism (e.g., [10], [21]–[25]). Mentalizing regions have also been found to support tasks other than those strictly pertaining to theory of mind. This includes awareness of the appropriateness of others' facial expressions in context [26], irony understanding during mismatches between words and context [27], moral judgments [28]–[30], empathy [31], emotional self-assessment [32], and violations of social norms [33], [34]. Some research has indicated that these mentalizing brain regions show reduced specialization in autism, even during tasks that do not strictly require mentalizing per se [27], [32]. Taken together, these findings in people with typical development and with autism suggest that the mentalizing network might also underlie additional tasks that show behavioral deficits in children with autism, such as identifying good and bad behaviors in others.

The purpose of the current study was to use fMRI to examine the brain mechanisms that underlie the previous reports of unusual behaviors of children with autism when making social judgments. More specifically, we wanted to elucidate the cognitive processes that were being used by the children with autism and the children with typical development when making these types of judgments by examining the concurrent patterns of brain activation. We created a nonverbal task in which children with autism and typical development viewed a pair of images and had to make either a social judgment or a physical judgment. We elected to do a physical judgment task for comparison because children with autism do not typically show deficits in these tasks behaviorally [35], [36], suggesting preserved ability. We hypothesized that the children with autism would show an unusual pattern of brain activity during the social judgment task, given the aforementioned deficits on other social tasks, but relatively similar activity while making physical judgments. Further, we investigated viewing patterns of the stimuli with eyetracking to determine whether the two groups of participants used the same visual information to make decisions. As required for interpretation of fMRI data, the task was designed so that all of the children were able to do it well; thus, any differences in brain activity could be attributed to neural strategy rather than performance. Another advantage of this task was that it did not inherently require a large amount of linguistic processing, making it possible to examine cognitive processing when making a social judgment alone rather than including the translation of that process into spoken language.

## Methods

### Participants

Participants' guardians provided written consent and participants provided written assent for this study, which was approved by the Institutional Review Boards at Carnegie Mellon University, the University of Pittsburgh, and Duquesne University. Participants were recruited from the pool of participants maintained by the Autism Center for Excellence Subject Core at the University of Pittsburgh; initial diagnostic and characterization testing of participants was performed by the Subject Core to establish eligibility for participation in research studies. Twelve children with autism (ages 8 to 16 years) and thirteen typically developing children (ages 7 to 15 years) were included in all the data analyses for this fMRI study. The two groups did not significantly differ in age and Full Scale, Verbal or Performance IQ (p>.05), as determined by administration of the Wechsler Abbreviated Scales of Intelligence (WASI; [37]). (See Table 1 for participant details.) An additional five children with autism and nine children with typical development were scanned but excluded from analyses due to low task performance (three with autism, one with typical development) or excessive head motion.

**Table 1:** Demographic information for autism and control groups.

		Autism (n = 12)	Control (n = 13)	*t* value	*P* 1
Age (years)	Mean ± SD	13.08±2.39	11.46±2.63	0.99	0.33
Verbal IQ	Mean ± SD	111.42±15.58	116.00±10.68	0.86	0.40
Performance IQ	Mean ± SD	110.67±15.51	113.85±10.14	0.61	0.55
Full-scale IQ	Mean ± SD	112.08±15.19	116.62±10.28	0.88	0.39
Handedness	R∶L∶A; Mean ± SD	10:1:1 (.67±.65)	13:0 (.98±.06)	1.74	0.10
Gender	Male∶Female	9:3	11:2		0.65
ADOS Communication	Mean ± SD	3.83±.94	N/A		
ADOS Social	Mean ± SD	7.25±1.22	N/A		
ADOS Total Soc Comm	Mean ± SD	11.08±1.31	N/A		

1 *p* value for gender was computed using the Fisher exact statistic; other *p* values were computed from the two-sample *t* test.

Autism diagnoses were based on the Autism Diagnostic Interview—Revised [38], [39], the Autism Diagnostic Observation Schedule [40], and expert clinical opinion. Potential participants with autism were excluded from recruitment into the larger participant pool if they had an identifiable cause for their autism, such as Fragile X syndrome, fetal cytomegalovirus infection, or tuberous sclerosis, or if they were found to have evidence of prematurity, head injury, birth asphyxia, or a seizure disorder. Exclusions were based on neurologic history and examination, physical examination, and chromosomal analysis or metabolic testing, if indicated. Potential control participants were screened by questionnaire, telephone, face-to-face interview, and observation during initial testing and were excluded if they had a current or past history of prematurity, birth injury, developmental delay, psychiatric or neurologic disorders, school problems, learning disabilities, acquired brain injury, or medical disorders with implications for the central nervous system. Exclusionary criteria for controls also included a history in first degree relatives of autism, developmental cognitive disorder, learning disability, schizophrenia, obsessive compulsive disorder, anxiety disorder, affective disorder, or other neurologic or psychiatric disorder thought to have a genetic component. Handedness was determined through administration of the Edinburgh Handedness Inventory [41]. All had normal or corrected-to-normal vision.

### Scanning

This block design fMRI experiment was performed using a 3 Tesla Siemens Allegra head-only scanner with 50-mT/m gradients (Siemens, Erlangen, Germany). Parallel imaging was performed using an 8-channel head coil. Participants' heads were immobilized using tape and foam pillows, and all participants underwent a preliminary practice session in a mock scanner to ensure that they understood how to remain still. Two hundred twenty-four T1-weighted anatomical images were acquired using an MPRAGE sequence (TR = 1630 ms, TE = 2.48 ms; FOV = 20.4 cm; α = 8°; image matrix = 2562; voxel size = 0.8×0.8×0.8 mm) to be later used for coregistration with the functional data. These structural images were aligned in the near-axial plane defined by the anterior and posterior commissures. Co-planar, whole-brain functional images were acquired using an echoplanar imaging sequence sensitive to blood oxygenation level dependent (BOLD) contrast (TR = 1000 ms; TE = 25 ms; α = 60°; FOV = 200 mm; image matrix = 642; voxel size 3.1×3.1×5 mm; 20 axial slices). Two discarded RF excitations ensuring steady-state equilibrium preceded the collection of 393 successive brain volumes.

Image preprocessing was performed in SPM 2. Images were corrected for slice acquisition timing and motion, normalized to the Montreal Neurological Institute (MNI) space, isovoxeled, and smoothed with an 8 mm Gaussian kernel to reduce noise. Individual and group analyses were performed using a general linear model and Gaussian random field theory [42]. For the random-effects group analyses, statistical maps were overlaid on normalized T1-weighted images. A threshold of t = 4.01 (p = .001) was used for within-group analyses and a threshold of t = 2.81 (p = .005) was used for between-group analyses, all with a minimum extent of six voxels.

### Stimuli

Participants viewed stimuli on a projection screen inside of the scanner using a mirror placed above the eyes. For each of the 32 trials, participants viewed two images from the “Goofus and Gallant” cartoons previously published in *Highlights for Children* [43] that had been provided to us and used with the permission of that magazine. Children were instructed to attend to the blond-haired boy in each image. In half of the trials, they were asked in which picture the blond-haired boy was being bad, or doing something that he was not supposed to do (Social condition). In the other half, the children had to indicate which picture took place outside (Physical condition). A symbol (a red circle with diagonal line through it for “bad” or a sun for “outside”) appeared at the beginning of a block to signal what type of question was to be answered. Then, two images were displayed for 4,000 ms. Next, the symbol appeared again below the two pictures with pictures of two hands on separate computer mice to signal that it was time to respond while the pictures remained onscreen for another 4,000 ms. Trials were organized into eight blocks, four of Social choices and four of Physical choices. The blocks were presented in sets of two for each condition to reduce the effects of task switching. All stimuli were counterbalanced for left and right answer choices. Paired frames were matched for the number of faces visible. Each block contained an instruction slide that was presented for 3,750 ms, four trials for a total of 32,000 ms, and four interstimulus pauses of 750 ms. A 16,000-ms fixation occurred between blocks. The total scan time was approximately 8.5 minutes.

Before the block presentation in the scan session, participants underwent an instruction and practice session at the beginning of the scan for 48,000 ms. They also performed a preliminary practice session of three Social and three Physical trials outside the scanner. Experimenters ensured that the children understood and could adhere to the instructions during the preliminary practice session outside of the scanner. As described earlier, three additional participants with autism and one with typical development, who were unable to perform the task with accuracy at or above 70%, were not included in any analyses.

### Eyetracking

Outside of the scanner, 14 of the children (7 with autism, 7 typically developing) who were scanned successfully participated in an eyetracking session, along with 6 (3 from each group) who were scanned unsuccessfully (i.e., their fMRI data was not usable because of excessive head motion or failure to complete the paradigm). Eyetracking occurred after the fMRI session. This follow-up study was performed to ensure that differences in brain activity could not be attributed to differences in viewing patterns. The stimuli were a subset consisting of 16 of the stimuli (8 Social, 8 Physical) used in the scanning session. Stimuli were presented using Tobii ClearView Software (Tobii Technology, Danderyd, Sweden) on a Tobii T60 eyetracking system (Tobii Technology, Danderyd, Sweden) with a 17” monitor. This paradigm consisted of two Social blocks alternated with two Physical blocks. Each block consisted of four trials and was presented as follows: 4,000 ms original frame presentation, 4,000 ms display of the frames with the pictures of the hands on the computer mice at the bottom, then 2,000 ms fixation. Each stimulus image was shown at 32 degrees of visual angle horizontally and 24 degrees vertically. Experimenters manually recorded the children's answers. For analysis, ROIs were drawn on the images. These were created for each individual face and body in the picture as well as for additional objects that were critical for determining behavior or otherwise potentially interesting. The location, number, and duration of fixations were recorded and analyzed.

## Results

### Behavioral performance

We performed repeated-measures ANOVAs to examine performance differences between the two groups. There was no difference in overall accuracy between the groups [F(1,23) = .00, p = .990], but there was a main effect of condition [F(1,23) = 38.87, p<.001]; both groups were more accurate in the Physical condition than in the Social condition. There was no significant interaction between group and condition [F(1,23) = 1.75, p = .199]. For the reaction time, there was no difference between the groups [F(1,23) = .442, p = .513], but there was a main effect of condition [F(1,23) = 64.51, p<.0001]; both groups were faster to respond in the Physical condition. Again, no significant interaction between group and condition was identified [F(1,23) = .199, p = .660].

### fMRI

For a list of all peaks in activity, see Tables S1 and S2.

#### Typically developing group

**Figure 1:**
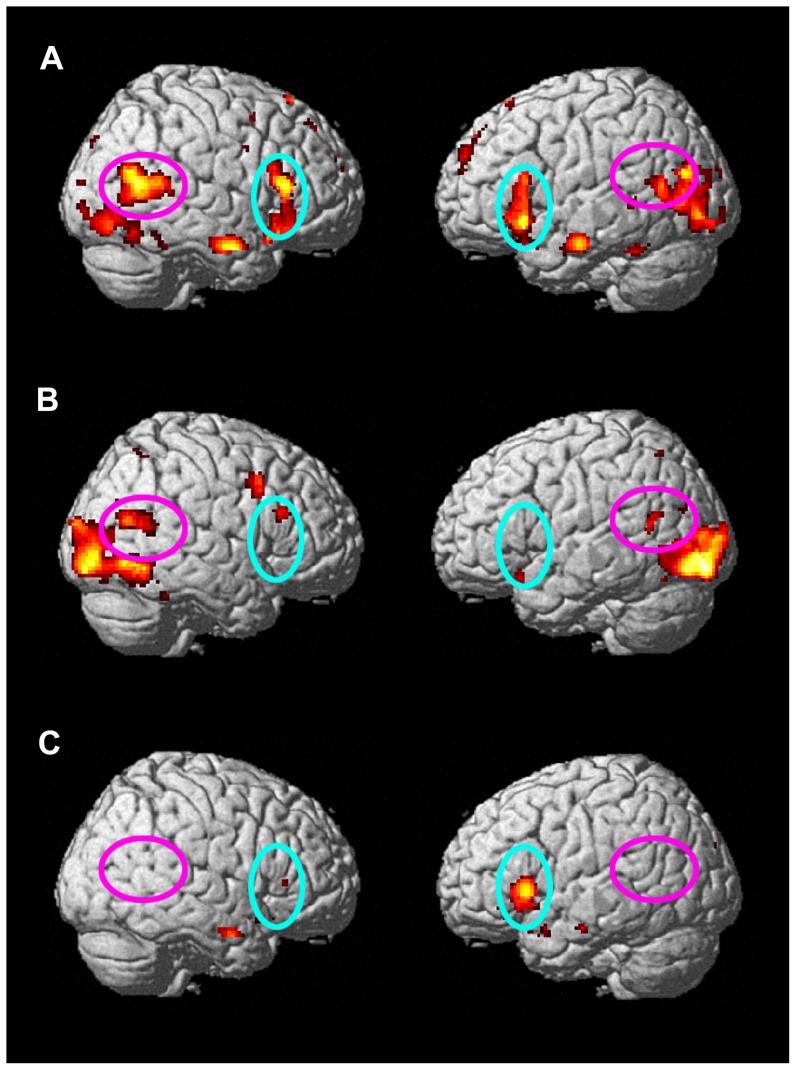
Activation maps for (a) Typically developing (TD) participants: Social – Physical; (b) Participants with Autism (AD): Social – Physical; and (c) TD – AD, Social – Physical. The IFG is circled in aqua, and the pSTS is circled in magenta.

All reported within-group comparisons were significant at p<.001. When Physical and Social conditions were combined and compared to fixation, there was increased activity in bilateral pars triangularis extending into pars opercularis (including Broca's area), middle frontal gyrus (MFG), occipital cortex extending into pSTS, and supplementary motor area (SMA); left inferior and middle temporal gyrus (ITG/MTG) and left precentral gyrus. When Physical alone was compared to fixation, there was increased activity in bilateral IFG including pars triangularis (Broca's area), insula, SMA, and MFG; left pre- and postcentral gyri and Rolandic operculum; and right putamen and superior frontal gyrus (SFG). Greater activity was found in response to Social than to fixation in bilateral occipital cortex into pSTS, Broca's area, MFG, and SMA; left caudate, MTG, precentral gyrus, superior medial frontal cortex, and thalamus; and right SFG. The Physical condition elicited a greater response than the Social condition in bilateral middle cingulum, Rolandic operculum, and SMA; left inferior parietal lobe (IPL), MFG, and pre- and postcentral gyri; and right precentral gyrus. The reverse comparison, where Social was greater than Physical, showed higher activity in bilateral Broca's area, superior medial frontal gyrus (medial frontal cortex), fusiform gyrus (FFG), MTG into pSTS, caudate, hippocampus, occipital cortex, and precuneus; and right cingulum, MFG, and precentral gyrus (Figure 1A).

#### Autism group

The combined response to Physical and Social conditions resulted in increased bilateral MFG, occipital cortex (extending into pSTS only in the right hemisphere), precuneus, and SMA; left amygdala, aMTG, caudate, and pre- and postcentral gyri; and right IFG, SFG, and superior parietal lobule (SPL). The Physical condition response was greater than that to fixation in bilateral MFG, occipital cortex (into right pSTS only), pre- and postcentral gyrus, SPL, and SMA; left amygdala, caudate, MTG, putamen, and Rolandic operculum; and right middle cingulum, parahippocampal gyrus, precuneus, SFG, and thalamus. When comparing the Social condition to fixation, activity was greater in the bilateral occipital cortex (extending into bilateral pSTS), precuneus, SMA, hippocampus, and insula; left amygdala, operculum, and temporal pole; and right pars triangularis, precentral gyrus, putamen, SFG, and SPL. The response to Physical was greater than to Social in bilateral cingulum, insula, postcentral gyrus, precentral gyrus, SMA, and supramarginal gyrus; and left IPL and MFG. The Social condition elicited a greater response than the Physical condition in bilateral occipital cortex, pSTS/MTG, and SPL; left FFG, insula, and superior temporal pole; and right MFG and pars triangularis within the IFG (Figure 1B).

#### Group comparisons

All reported results for the between-group comparisons were significant at p<.005. For the combined conditions greater than fixation comparison, the response in the typical development group was greater than that of the autism group in bilateral IFG, right MFG, and left insula. The autism group showed a greater response than the typically developing group in this comparison in bilateral occipital cortex, right STG, and right middle cingulum. When the Physical condition was compared to fixation, the typical development group demonstrated higher levels of activity than the autism group in the right precentral gyrus, right MFG and IFG at the pars orbitalis, and left SPL, and the autism group had higher activity than the typical development group in bilateral anterior cingulate and occipital cortex, and left MTG, amygdala, MFG, and an area of IFG at the pars orbitalis not seen in our other analyses. For Social greater than fixation, the typical development group showed greater activity than the autism group in bilateral IFG (pars triangularis and orbitalis) and insula; left superior temporal pole, MTG, pallidum, hippocampus, and caudate; and right MFG. The response in the autism group was greater than that of the typical development group in bilateral occipital cortex and right precentral and postcentral gyri, middle cingulum, STG, and MFG.

The typical development group had greater activity than the autism group to Physical than Social in the right MFG, inferior temporal gyrus (ITG), precentral gyrus, and SMA as well as left occipital cortex and IPL. (See Figure 1C.) For the Social greater than Physical comparison, our primary contrast of interest, the children with typical development showed greater responses than the children with autism in bilateral IFG at pars triangularis (Broca's area), aSTS/MTG, insula and anterior cingulate as well as left hippocampus, superior temporal pole, pallidum, cuneus, IFG at the pars orbitalis, and precuneus. There were no areas in which the brain activation of the autism group was greater than that of the group with typical development.

### Eyetracking results

Eyetracking was performed on 14 of the participants (seven from each group) who were successfully scanned as well as an additional six participants who were unsuccessfully scanned (three from each diagnostic group) on a subset of the paradigm stimuli outside of the scanner, for a total of ten eyetracking participants per group. There were no group differences for the percentage of task time spent focused on the entire task (including stimuli, instructions, and fixation slides) (mean typical development group = 96.49%, mean autism group = 94.5%, t(18) = 0.67, p = 0.51), the Physical stimuli (mean typical development group = 37.72%, mean autism group = 36.67%, t(18) = 1.21, p = 0.24), and the Social stimuli (mean typical development group = 38.15%, mean autism group = 37.70%, t(18) = 0.65, p = 0.52). The eyetracking results suggest that the fMRI activation findings cannot be attributed to overall attention to the stimuli and the task.

**Figure 2:**
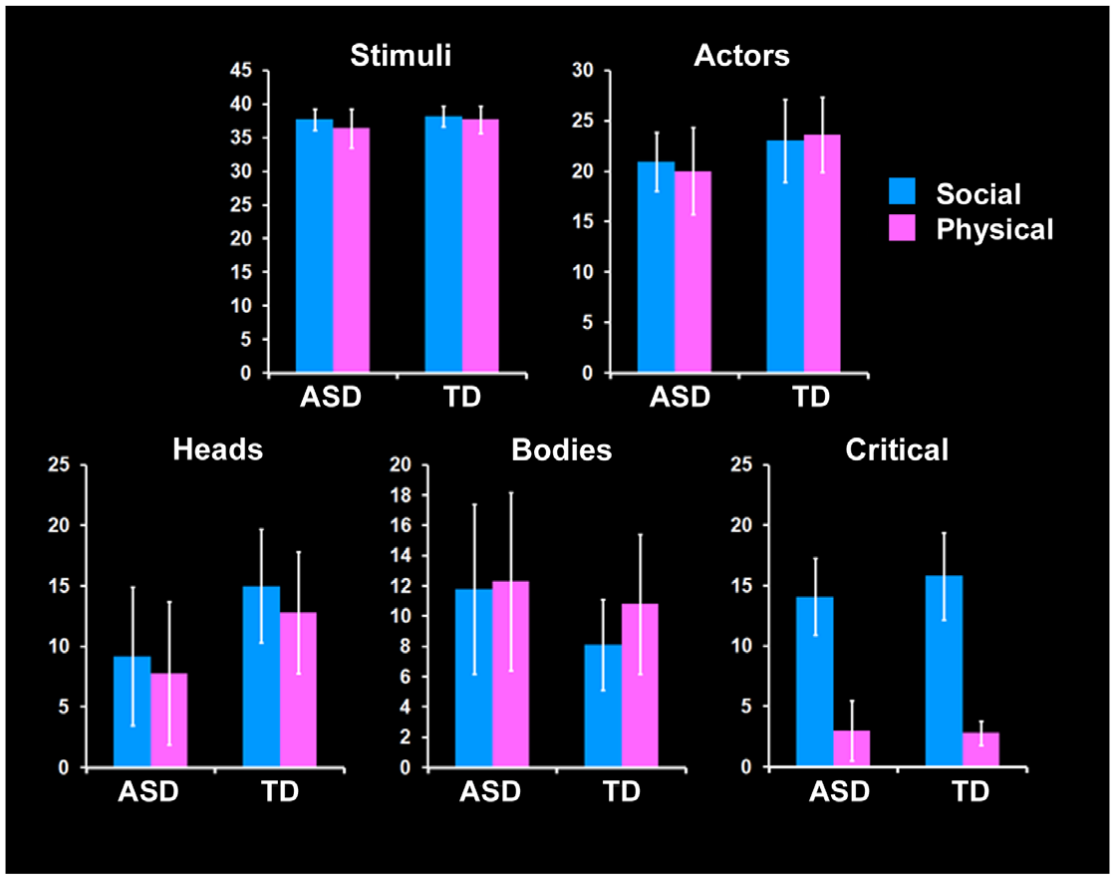
Eyetracking results. Graphs of the mean percentages of time the participants fixated on segments of the stimuli relative to the total amount of time they were present on the screen. Error bars denote standard deviations.

Next, we examined how much time the participants spent looking at the individuals in the images who were taking part in the action of the scene (henceforth called Actors). These calculations are reported as a percentage of the time during which stimuli were on the screen. The children with autism had a statistically non-significant trend towards spending less time viewing the Actors than the children with typical development did throughout the task (mean typical development group = 46.68%, mean autism group = 41.09%, t(18) = 1.83, p = 0.08). This appeared to arise from a difference in viewing patterns during the Physical stimuli (mean typical development group = 23.65%, mean autism group = 20.13%, t(18) = 1.96, p = 0.07) rather than the Social stimuli (mean typical development group = 23.03%, mean autism group = 20.96%, t(18) = 1.31, p = 0.21). Given that the Physical task can be done without reference to the individuals in the images, this suggests that the children with typical development may look more at the actors than the children with autism do when it is unnecessary to do so, although the difference was not statistically reliable. The proportion of stimulus viewing time spent on actors' heads was greater for the typical development group than the autism group for the full task (mean typical development group = 27.82%, mean autism group = 16.99%, t(18) = 2.32, p = 0.03) as well as the Physical and Social conditions (Physical: mean typical development group = 12.84%, mean autism group = 7.81%, t(18) = 2.05, p = 0.05; Social: mean typical development group = 14.98%, mean autism group = 9.19%, t(18) = 2.46, p = 0.02). No trends were found in the amount of time spent viewing actors' bodies for the full task or the Physical condition (p>0.20); however, there was a statistically non-significant trend towards greater viewing of actors' bodies by the autism group in the Social task (mean typical development group = 8.05%, mean autism group = 11.78%, t(18) = 1.85, p = 0.08). No differences were found between groups for the amount of time spent viewing critical regions—those necessary for determining the answer—in any condition (all p>0.25). (See Figure 2.)

We would predict that the viewing differences for the heads and bodies, if significantly driving brain responses, would be reflected in higher activity levels in the fusiform gyrus, particularly the fusiform face area (e.g., [44]) and fusiform body area [45], as well as the extrastriate body area [46]. However, between-group differences in these regions were not identified. Moreover, the differences that were identified were not in regions typically implicated in face processing, body processing, or overall attention. Together, these results suggest that the differences in brain activity are not likely to be a result of differential viewing patterns between the two groups of children.

## Discussion

When performing social versus physical judgments, the children with typical development had relatively higher levels of brain activity in social cognition and language areas, including bilateral mPFC (within the medial frontal cortex, MFC), IFG, FFG, and pSTS. However, the children with autism did not show this same pattern of increased activity in the mPFC, left IFG, or left FFG, despite similar activity to that seen in the children with typical development in subregions of right IFG, right FFG, and bilateral pSTS. The group with typical development had a significantly stronger differential response to the social scenarios than the group with autism in a subset of social and language areas, including the mPFC, left superior temporal pole, bilateral IFG at the pars triangularis, and the left IFG at the pars orbitalis. These differences occurred despite similar accuracy in identifying the inappropriate social behavior in both groups; therefore, the differences in activation did not result from differences in the accuracy of responses. In addition, the results of a follow-up eyetracking study suggest that the activation differences are not likely being driven purely by different gaze patterns between groups. The typically developing group did have greater looking time to faces than the autism group, but the activation differences were not in areas traditionally associated with face processing.

Overall, the fMRI findings indicate that a reduced subset of the mentalizing and language networks is sufficient for accurate performance on this relatively easy set of social and physical judgment tasks and that this functioning is preserved in children with autism, with typically developing children recruiting more brain regions than are strictly necessary to accomplish the processing task. Thus, the children with autism appear to have used a neural strategy that was a subcomponent of that used by the children with typical development rather than a novel, alternative strategy. The additional brain regions recruited by the children with typical development are ones that represent traditional social and language areas, suggesting that, unlike the children with autism, the children with typical development were automatically encoding their social knowledge into language. Although activity patterns were similar in the groups with typical development and autism in some social brain regions, different patterns of processing were observed in others, including the mPFC, left superior temporal pole, and bilateral IFG. The MFC, which contains the mPFC, has been implicated in social cognition broadly as well as mentalizing and has been functionally divided into posterior rostral (pr), anterior rostral (ar), and orbital subregions (o) (for review, see [47]). We found differences across groups between the Social and Physical conditions in the arMFC. This area of the MFC has been implicated in theory of mind (e.g., [19], [21], [48]–[50]), empathy [31], violations of social norms [33], [34], morality (e.g., [28]–[30]; for review, see [51]), appropriateness of facial expressions [26], and person perception and monitoring [52]–[54]. Moreover, it has previously been shown to have unusual activity patterns during tasks requiring theory of mind in adults with autism [21], [55] and irony comprehension in children with autism [27]. It has been suggested that the regional cerebral blood flow is abnormal in general for medial prefrontal cortex in autism, and that this correlates with communication and social interaction scores [56]. We provide further evidence of abnormal activity patterns in this region in children with autism that are consistent with the previous research in both adults and children; at the same time, we provide additional understanding of the role of this region in a new task of social judgment.

In addition to the social and language regions about which we had hypotheses, the left superior temporal pole showed differences between the two groups. In typically developing individuals, the temporal poles have been implicated in a number of social processes, including mentalizing (e.g., [19], [31], [48]–[50], [57]), empathizing [31], inferring the emotional states of others, and understanding socially important narratives (for review, see [58]). The left temporal pole is involved in moral judgments [28], [59] and analyzing the appropriateness of facial expressions in context [26]. Finally, the bilateral temporal pole is also more active when reading stories that involve social norm violations relative to normal stories, suggesting that this region is involved in analyzing the appropriateness of behavior [33]. Taken together, these and other results have been interpreted to mean that the temporal poles play an important role in making inferences about other people's feelings and behavior, perhaps by coupling high-level perception with personal visceral emotion and semantic information [58]. Abnormal temporal pole activity has been found in adults with autism, and it has been suggested that the temporal pole is a component of a key neural circuit that is impaired in autism [60]. Additionally, the temporal pole does not deactivate after resting state in individuals with autism, despite doing so in typically developing individuals [61]. Recently, it has been shown that bilateral temporal pole activity is abnormal when comparing the response to happy and neutral faces in individuals with autism as well as their siblings relative to typically developing individuals [62]. Our findings that the group with typical development recruited the left superior temporal pole for the social condition more than the group with autism also suggests a role for the temporal pole in making simple social judgments in typically developing individuals and provides further evidence for abnormal function in this region in autism.

We also found differences in the activity patterns in both right and left IFG. In typically developing individuals, right IFG has been implicated as a mirror neuron region in which the neurons fire both when the individual performs an action and when the individual sees another person performing the same action (for review, see [63]). Reduced activity in this area has been reported for individuals with autism during social tasks (e.g., [64]). In children with autism, similar findings have been reported for social tasks (e.g., [65]) and social language processing [66]. Interestingly, relatively increased activity in right IFG has been reported for children with autism during irony comprehension, a result that was interpreted as representing an increase in effortful processing and possible use of a compensatory strategy [27]. Thus, our finding that children with autism show reduced differentiation in right IFG activity relative to typically developing children is unsurprising; however, it is difficult to tease apart the separate roles of linguistic and social processing in the task used in the current study.

Left IFG is a language processing region, and several studies have reported relatively reduced activation in this region in adults with autism during language tasks (e.g., [67], [68]). In the current study, the relatively increased left IFG activation by the children with typical development suggests that they were using language processing even though the social judgment task did not explicitly require them to do so. Prior fMRI research has found similar differences in the use of language in individuals with autism, with the comparison group activating language regions whereas the autism group did not, even though they successfully performed the task. For example, individuals with autism were reported to be less likely to recruit brain regions involved in verbal processes during memory tasks for letters [69] and faces [70]. Although verbal processing is not strictly necessary for either task, typically developing individuals recruited left IFG, whereas participants with autism did not. Sahyoun and colleagues [71] used a picture task where participants saw three images in a grid and had to determine the fourth image from a set of choices. For each grid, one of three types of strategies was possible: visuospatial, semantic, or a hybrid visuospatial and semantic method. The typically developing children used left IFG whenever semantic strategies were available or necessary, whereas the children with autism relied on posterior regions for all strategies. Additionally, the children with autism showed reduced fronto-temporal connectivity. These studies suggest that there is automaticity of verbal encoding in typically developing individuals that is reduced or absent in individuals with autism.

A tendency not to automatically recruit linguistic encoding could explain some of the previous behavioral results in which children with autism were less accurate in identifying inappropriate verbal behaviors [11] and offered bizarre explanations for their responses [11], [14]. Both research teams suggested that abnormal language processing could be responsible for poor performance [11], [14]. Moreover, it is in line with anecdotal reports and case studies from individuals with autism that they primarily rely on nonverbal thought (e.g., [72]). For example, Temple Grandin described her life with autism:

“I think in pictures. Words are like a second language to me. I translate both spoken and written words into full-color movies, complete with sound, which run like a VCR tape in my head. When somebody speaks to me, his words are instantly translated into pictures” ([73], pg. 1).

Our findings provide further support that individuals with autism, unlike typically developing individuals, do not automatically use verbal strategies to complete tasks in which they are not strictly necessary. That is, linguistic skills are not immediately brought on line to assist with cognitive processing tasks. This tendency can help explain the poorer performance seen in individuals with autism on social judgment tasks that require linguistic knowledge to respond accurately. It also helps to clarify how accuracy is preserved for some tasks that do not require language, while verbal justification remains poor.

This finding is particularly interesting in light of Gazzaniga's theory of language function derived from research on typically developing individuals and split-brain patients. He proposed that left hemisphere language regions (“the interpreter”) are automatically engaged to interpret stimuli and assimilate them into comprehensible events (e.g., [74]). This automatic self-storytelling allows for elaboration and generalization of information such that the left hemisphere is “drive[n] to create order from apparent chaos” by providing a narrative and relating information to other remembered events while the right hemisphere maintains a more veridical record of events ([74], pg. 1319). Reports of split-brain patients have supported this role of the left hemisphere in finding patterns among stimuli (e.g., [75]). We hypothesize that this function might be disrupted in autism spectrum disorders such that no automatic storytelling or conversion of information into linguistic form occurs, as evidenced by the reduced left IFG activity found in this study. A disrupted tendency to create narratives could result in reduced organization and comprehension of incoming stimuli and a lower ability to generalize across situations because of reduced use of linguistic forms to promote integration of past knowledge. The lack of a left hemisphere “interpreter” could account for the poor behavioral performance in identifying and explaining behavioral judgments previously reported [11], [14].

The failure of individuals with autism to recode experiences into language or to use language as a scaffold has been previously proposed as an underlying cause for difficulty with recalling experiences [76] and for difficulty with recalling the information presented in pictures of everyday family scenes [77]. The lack of automatic storytelling on the part of the children with autism may explain both the necessity for and the success of the use of intervention strategies such as social stories [78], [79]. These interventions do the work, in effect, that the brains of the children with autism are not doing. Interestingly, the results in language regions suggest that the storytelling process is more reduced in autism relative to typical development for social than for nonsocial judgments. These findings are consistent with the results reported by Pierce and colleagues [9] in which children with autism performed as well as verbal mental-aged matched and age-matched children on answering factual questions but not social questions. Future research is necessary to clarify which domains are affected by automatic storytelling differences and whether individuals with autism might show preserved or even enhanced automatic storytelling in non-social tasks. Specifically, automatic social storytelling could be uniquely affected because of an absence of all automatic social processing in non-explicit tasks (e.g., [80], [81]).

In conclusion, we find that children with autism do not recruit the same network of social and language regions as typically developing children when performing a social judgment rather than a physical judgment, despite similar behavioral performance. Instead, the children with autism rely on a subset of the brain regions used by children with typical development. This has two major implications. First, it provides further evidence that typically developing children recruit additional social and language regions for relatively simple judgments, even when they are not strictly required for task performance. These findings also provide an interesting addition to previous reports that typically developing adults recruit more language areas even when a given task does not necessitate it for performance, showing a preference for linguistic over visual strategies [69]–[71]. This preference towards using a more complex strategy may thus be extended to the social domain. Second, it suggests that abnormal functioning in the IFG, mPFC, and temporal poles in autism does not correspond to inferior task performance, which is particularly important for understanding the roles of these areas in social cognition in autism. The previous reports of decreased behavioral performance on social judgment tasks by individuals with autism (e.g., [9], [11], [14]) could be accounted for by the increased difficulty inherent in these tasks, including verbalization of decision criteria, which may result in the need for these regions. Children with autism may recognize socially inappropriate behavior but may not automatically use language to encode this understanding, making expression and generalization of this knowledge more difficult for them. Future research could address this issue by examining the interaction of task difficulty and performance with brain activity on this task in individuals with and without autism.

## Supporting Information

Table S1 **Peaks of activity for the within-groups contrasts, p<.001.**
(DOC)

Table S2 **Peaks of activity for the between-groups contrasts, p<.005.**
(DOC)
