# Supplementary material for: Is He Being Bad? Social and Language Brain Networks during Social Judgment in Children with Autism
Source: PLoS One. 2012 Oct 17;7(10):e47241. doi: 10.1371/journal.pone.0047241 (PMC3474836; doi:10.1371/journal.pone.0047241)
Supplement: Table S1 — Peaks of activity for the within-groups contrasts, p<.001. (DOC) [file pone.0047241.s001.doc]

Supplementary Table S1. Peaks of activity for the within-groups contrasts, p < .001.

|  |  |  | | |  |  | | |
| --- | --- | --- | --- | --- | --- | --- | --- | --- |
|  |  |  | | |  |  | | |
|  |  |  | | |  |  | | |
|  |  |  | MNI Coordinates | | | |  | |
| *Typical Development All > Fixation* | Hemi. | X | | | Y | Z | | |
| Inferior and middle temporal gyri | L | -62 | | | -6 | 28 | | |
| Inferior frontal gyrus–triangularis | R | 56 | | | 30 | 4 | | |
| Inferior frontal gyrus–triangularis, orbitalis* | L | -28 | | | 22 | -6 | | |
| Inferior frontal gyrus–triangularis, orbitalis | R | 26 | | | 20 | 0 | | |
| Middle frontal gyrus | R | 40 | | | 4 | 58 | | |
| Middle frontal gyrus | R | 52 | | | 16 | 46 | | |
| Occipital cortex | R, L | 4 | | | -98 | -2 | | |
| Precentral gyrus | L | -36 | | | -2 | 64 | | |
| Precentral gyrus | L | -40 | | | -14 | 64 | | |
| Supplementary motor area | R, L | 2 | | | 18 | 50 | | |
| Supplementary motor area | R | 12 | | | 10 | 72 | | |
| *Autism All > Fixation* |  |  | | |  |  | | |
| Amygdala | L | -20 | | | -2 | -22 | | |
| Caudate | L | -12 | | | 24 | 0 | | |
| Inferior and middle frontal gyri | R | 40 | | | 6 | 34 | | |
| Middle frontal gyrus | L | -36 | | | 2 | 50 | | |
| Middle temporal gyrus | L | -62 | | | -6 | 24 | | |
| Occipital cortex | R, L | 36 | | | -72 | -26 | | |
| Precentral and postcentral gyri | L | -40 | | | -14 | 66 | | |
| Precentral gyrus** | L | -52 | | | 14 | 36 | | |
| Precuneus | R, L | 0 | | | -52 | 52 | | |
| Superior frontal gyrus | R | 30 | | | -8 | 70 | | |
| Superior parietal lobule | R | 30 | | | -62 | 50 | | |
| Supplementary Motor Area | R, L | -10 | | | 14 | 48 | | |
|  |  |  | | |  |  | | |
|  |  |  | | |  |  | | |
|  |  |  | MNI Coordinates | | | | |  |
| *Typical Development Physical > Fixation* | Hemi. | X | | | Y | Z | | |
| Inferior frontal gyrus–opercularis | R | 42 | | | 20 | 32 | | |
| Inferior frontal gyrus–opercularis | L | -46 | | | 6 | 28 | | |
| Inferior frontal gyrus–triangularis | L | -58 | | | 24 | 22 | | |
| Inferior frontal gyrus–triangularis | R | 40 | | | 26 | 20 | | |
| Insula | L | -26 | | | 26 | 0 | | |
| Insula | R | 32 | | | -24 | 24 | | |
| Middle frontal gyrus | L | -48 | | | 32 | 36 | | |
| Middle frontal gyrus | R | 40 | | | 2 | 58 | | |
| Middle temporal gyrus | L | -58 | | | -50 | 2 | | |
| Occipital cortex | R, L | 2 | | | -98 | 4 | | |
| Paracentral lobule | R | 12 | | | -36 | 68 | | |
| Postcentral gyrus | L | -38 | | | -36 | 44 | | |
| Precentral gyrus | L | -34 | | | -4 | 66 | | |
| Precentral gyrus | L | -22 | | | -14 | 72 | | |
| Precentral gyrus | L | -58 | | | 10 | 34 | | |
| Putamen | R | 28 | | | 22 | 0 | | |
| Putamen | R | 30 | | | 0 | 6 | | |
| Rolandic operculum | L | -42 | | | -4 | 14 | | |
| Superior frontal gyrus | R | 24 | | | 2 | 72 | | |
| Supplementary motor area | R, L | -16 | | | 22 | 28 | | |
| *Autism Physical > Fixation* |  |  | | |  |  | | |
| Amygdala | L | -20 | | | -4 | -24 | | |
| Caudate | L | 2 | | | 6 | -14 | | |
| Middle cingulate | R | 14 | | | 18 | 42 | | |
| Middle frontal and precentral gyri | L | -48 | | | 6 | 52 | | |
| Middle frontal gyrus | R | 38 | | | 46 | 38 | | |
| Middle temporal gyrus | L | -62 | | | -6 | -24 | | |
| Occipital cortex | R, L | 20 | | | -104 | 8 | | |
| Parahippocampal gyrus | R | 24 | | | -16 | -20 | | |
| Precentral and postcentral gyri | L | -38 | | | -18 | 68 | | |
| Precentral and postcentral gyri | R | 48 | | | -18 | 62 | | |
| Precentral gyrus | R | 42 | | | 6 | 34 | | |
| Precuneus | R | 2 | | | -56 | 24 | | |
| Putamen | L | -32 | | | -2 | -6 | | |
| Rolandic operculum | L | -44 | | | -20 | 22 | | |
| Superior frontal gyrus | R | 30 | | | -10 | 72 | | |
| Superior parietal lobule | L | -30 | | | -70 | 46 | | |
| Superior parietal lobule | R | 26 | | | -58 | 48 | | |
| Supplementary motor area | R | 2 | | | 0 | 72 | | |
| Supplementary motor area | L | -10 | | | 14 | 48 | | |
| Thalamus | R | 20 | | | -10 | -2 | | |
|  |  |  | | |  |  | | |
|  |  |  | | |  |  | | |
|  |  |  | | MNI Coordinates | | | |  |
| *Typical Development Social > Fixation* | Hemi. | X | | | Y | Z | | |
| Caudate | L | -10 | | | 6 | 10 | | |
| Inferior frontal gyrus–opercularis | L | -42 | | | 4 | 26 | | |
| Inferior frontal gyrus–opercularis, triangularis, orbitalis | R | 36 | | | 26 | -18 | | |
| Inferior frontal gyrus–triangularis | L | -54 | | | 26 | 30 | | |
| Inferior frontal gyrus–triangularis, orbitalis* | L | -30 | | | 20 | -8 | | |
| Middle frontal gyrus | R | 40 | | | 6 | 54 | | |
| Middle frontal gyrus | R | 52 | | | 16 | 46 | | |
| Middle temporal gyrus | L | -64 | | | -8 | -28 | | |
| Occipital cortex | R, L | 2 | | | -98 | -2 | | |
| Precentral gyrus | L | -44 | | | 6 | 48 | | |
| Superior frontal gyrus | R | 34 | | | 0 | 68 | | |
| Superior medial frontal gyrus | L | -12 | | | 26 | 34 | | |
| Supplementary motor area | R, L | 10 | | | 24 | 38 | | |
| Thalamus | L | -12 | | | -14 | 14 | | |
| *Autism Social > Fixation* |  |  | | |  |  | | |
| Hippocampus | R | 20 | | | -8 | -16 | | |
| Hippocampus/amygdala | L | -16 | | | -6 | -14 | | |
| Inferior frontal gyrus–opercularis | L | -34 | | | 16 | 32 | | |
| Inferior frontal gyrus–triangularis | R | 40 | | | 6 | 36 | | |
| Insula | L | -30 | | | 20 | -6 | | |
| Occipital cortex | R, L | 38 | | | -52 | -20 | | |
| Precentral and inferior frontal gyri | L | -52 | | | 10 | 42 | | |
| Precentral gyrus | L | -30 | | | -10 | 70 | | |
| Precentral gyrus | L | -36 | | | 2 | 50 | | |
| Precuneus | R, L | 0 | | | -52 | 52 | | |
| Putamen and insula | R | 26 | | | 24 | 0 | | |
| Superior frontal gyrus | R | 38 | | | -10 | 68 | | |
| Superior parietal lobule | R | 20 | | | -64 | 60 | | |
| Supplementary motor area | R, L | -8 | | | 16 | 48 | | |
| Temporal pole | L | -42 | | | 20 | -20 | | |
|  |  |  | | |  |  | | |
|  |  |  | | |  |  | | |
|  |  |  | | MNI Coordinates | | | |  |
| *Typical Development Physical > Social* | Hemi. | X | | | Y | Z | | |
| Inferior parietal lobule | L | -54 | | | -50 | 52 | | |
| Inferior parietal lobule | L | -42 | | | -36 | 38 | | |
| Middle cingulum | L | -18 | | | -6 | 44 | | |
| Middle cingulum | R | 18 | | | -34 | 36 | | |
| Middle cingulum | L | -8 | | | -30 | 44 | | |
| Middle frontal gyrus | L | -46 | | | 48 | 10 | | |
| Postcentral and precentral gyri | L | -26 | | | -30 | 62 | | |
| Precentral gyrus | R | 44 | | | -14 | 46 | | |
| Rolandic operculum | L | -50 | | | 0 | 2 | | |
| Rolandic operculum | R | 44 | | | 2 | 10 | | |
| Supplementary motor area | L | -12 | | | -12 | 64 | | |
| Supplementary motor area | R | 16 | | | -12 | 66 | | |
| *Autism Physical > Social* |  |  | | |  |  | | |
| Cingulum | R, L | 14 | | | 2 | 40 | | |
| Cingulum | L | -6 | | | 12 | 38 | | |
| Inferior parietal lobule | L | -52 | | | -22 | 40 | | |
| Insula | L | -38 | | | -18 | 16 | | |
| Insula | R | 44 | | | 2 | -4 | | |
| Middle frontal gyrus | L | -42 | | | 52 | 14 | | |
| Postcentral gyrus | L | -18 | | | -38 | 66 | | |
| Precentral and postcentral gyri | L | -34 | | | -22 | 58 | | |
| Precentral and postcentral gyri | R | 26 | | | -32 | 70 | | |
| Precentral gyrus | R | 48 | | | -18 | 46 | | |
| Supplementary motor area | R, L | -14 | | | -4 | 44 | | |
| Supramarginal gyrus | R | 50 | | | -26 | 38 | | |
| Supramarginal gyrus | L | -54 | | | -30 | 24 | | |
|  |  |  | | |  |  | | |
|  |  |  | | |  |  | | |
|  |  |  | MNI Coordinates | | | | |  |
| *Typical Development Social > Physical* | Hemi. | X | | | Y | Z | | |
| Caudate | R, L | -6 | | | 6 | 6 | | |
| Cingulum | R | 12 | | | 32 | 32 | | |
| Fusiform gyrus | L | -40 | | | -44 | -24 | | |
| Fusiform gyrus | R | 44 | | | -46 | -22 | | |
| Hippocampus | L | -28 | | | -28 | -6 | | |
| Hippocampus | R | 28 | | | -28 | -6 | | |
| Inferior frontal gyrus–triangularis, orbitalis | R | 34 | | | 16 | -18 | | |
| Inferior frontal gyrus–triangularis, orbitalis* | L | -30 | | | 18 | -8 | | |
| Middle frontal gyrus | R | 42 | | | 6 | 52 | | |
| Middle temporal gyrus | R | 52 | | | -2 | -26 | | |
| Middle temporal gyrus | R | 52 | | | -56 | 10 | | |
| Middle temporal gyrus | L | -60 | | | -12 | -20 | | |
| Middle temporal gyrus | L | -50 | | | -44 | 2 | | |
| Middle temporal gyrus | L | -62 | | | -56 | 16 | | |
| Occipital cortex | L | -52 | | | -76 | 14 | | |
| Occipital cortex | R | 28 | | | -56 | -10 | | |
| Occipital cortex | R | 34 | | | -94 | 14 | | |
| Occipital cortex | R | 36 | | | -94 | -2 | | |
| Occipital cortex | R | 28 | | | -86 | 40 | | |
| Occipital cortex | L | -18 | | | -96 | -4 | | |
| Occipital cortex | L | -26 | | | -96 | 20 | | |
| Precentral gyrus | R | 40 | | | 4 | 30 | | |
| Precuneus | R, L | 2 | | | -54 | 26 | | |
| Superior medial frontal gyrus | R, L | -4 | | | 46 | 16 | | |
| Superior medial frontal gyrus | R, L | 6 | | | 30 | 64 | | |
| Superior medial frontal gyrus | R | 8 | | | 64 | 6 | | |
| *Autism Social > Physical* |  |  | | |  |  | | |
| Fusiform gyrus | L | -28 | | | -48 | -12 | | |
| Inferior frontal gyrus–triangularis | R | 34 | | | 20 | 18 | | |
| Insula | L | -26 | | | 24 | 0 | | |
| Middle frontal gyrus | R | 40 | | | 10 | 40 | | |
| Middle temporal gyrus | R, L | 44 | | | -56 | 16 | | |
| Middle temporal gyrus | L | -52 | | | -58 | 18 | | |
| Occipital cortex | R | 46 | | | -56 | -10 | | |
| Occipital cortex | L | -28 | | | -92 | -4 | | |
| Occipital cortex | L | -26 | | | -72 | 26 | | |
| Occipital cortex | L | -48 | | | -64 | -18 | | |
| Occipital cortex | R | 30 | | | -68 | 32 | | |
| Superior parietal lobule | L | -22 | | | -62 | 56 | | |
| Superior parietal lobule | R | 28 | | | -54 | 54 | | |
| Superior temporal pole | L | -42 | | | 20 | -18 | | |
|  |  |  | | |  |  | | |
| *extends into superior temporal pole |  |  | | |  |  | | |
| *extends into MFG, IFG |  |  | | |  |  | | |
