# Supplementary material for: Is He Being Bad? Social and Language Brain Networks during Social Judgment in Children with Autism
Source: PLoS One. 2012 Oct 17;7(10):e47241. doi: 10.1371/journal.pone.0047241 (PMC3474836; doi:10.1371/journal.pone.0047241)
Supplement: Table S2 — Peaks of activity for the between-groups contrasts, p<.005. (DOC) [file pone.0047241.s002.doc]

Supplementary Table S2. Peaks of activity for the between-groups contrasts, p < .005.

|  |  |  | | |  |  | | | |
| --- | --- | --- | --- | --- | --- | --- | --- | --- | --- |
|  | |  | | |  |  | | | |
|  |  |  | | |  |  | | | |
|  |  |  | | MNI Coordinates | | | |  | |
| *Typical Development > Autism, All > Fixation* | Hemi. | X | | | Y | Z | | | |
| Inferior frontal gyrus–opercularis | L | -48 | | | 12 | 4 | | | |
| Inferior frontal gyrus–orbitalis | R | 44 | | | 42 | -12 | | | |
| Inferior frontal gyrus–orbitalis, triangularis | L | -46 | | | 36 | -2 | | | |
| Insula | L | -28 | | | 24 | -2 | | | |
| Middle frontal gyrus | R | 42 | | | 4 | 58 | | | |
| Middle frontal gyrus–orbitalis | R | 30 | | | 48 | -12 | | | |
| *Autism > Typical Development, All > Fixation* |  |  | | |  |  | | | |
| Inferior occipital gyrus | L | -26 | | | -88 | -4 | | | |
| Middle cingulum | R | 6 | | | -36 | 48 | | | |
| Superior occipital gyrus | R | 22 | | | -84 | 14 | | | |
| Superior occipital gyrus | R | 18 | | | -100 | 18 | | | |
| Superior occipital gyrus | L | -14 | | | -98 | 26 | | | |
| Superior temporal gyrus | R | 46 | | | -46 | 0 | | | |
|  |  |  | | |  |  | | | |
|  |  |  | | |  |  | | | |
|  |  |  | | MNI Coordinates | | | |  | |
| *Typical Development > Autism, Physical > Fixation* | Hemi. | X | | | Y | Z | | | |
| Inferior and middle frontal gyrus–orbitalis | R | 44 | | | 44 | -10 | | | |
| Superior parietal lobule | L | -22 | | | -52 | 48 | | | |
| Precentral gyrus | R | 22 | | | -16 | 68 | | | |
| *Autism > Typical Development, Physical > Fixation* |  |  | | |  |  | | | |
| Amygdala | L | -22 | | | -2 | -24 | | | |
| Anterior Cingulum | R, L | 4 | | | 24 | -6 | | | |
| Inferior frontal gyrus–orbitalis | L | -36 | | | 34 | -6 | | | |
| Middle frontal gyrus | L | -38 | | | 10 | 52 | | | |
| Middle temporal gyrus | L | -60 | | | -4 | -22 | | | |
| Superior occipital gyrus | R, L | 22 | | | -86 | 16 | | | |
| Superior occipital gyrus | L | -14 | | | -98 | 26 | | | |
|  |  |  | | |  |  | | | |
|  |  |  | | |  |  | | | |
|  |  |  | | | MNI Coordinates | | |  | |
| *Typical Development > Autism, Social > Fixation* | Hemi. | X | | | Y | Z | | | |
| Caudate | L | -12 | | | -6 | 24 | | | |
| Hippocampus | L | -32 | | | -16 | -16 | | | |
| Inferior frontal gyrus–orbitalis* | L | -32 | | | 16 | -22 | | | |
| Inferior frontal gyrus–orbitalis, triangularis | L | -52 | | | 18 | -2 | | | |
| Inferior frontal gyrus–orbitalis, triangularis | R | 44 | | | 28 | 4 | | | |
| insula | R | 30 | | | 14 | -18 | | | |
| Insula | L | -28 | | | 20 | -6 | | | |
| Middle frontal gyrus | R | 42 | | | 4 | 58 | | | |
| Middle temporal gyrus | L | -64 | | | -50 | -4 | | | |
| Middle temporal gyrus | L | -68 | | | -42 | -2 | | | |
| Pallidum | L | -14 | | | 4 | 2 | | | |
| Superior temporal pole | L | -44 | | | 10 | -24 | | | |
| *Autism > Typical Development, Social > Fixation* |  |  | | |  |  | | | |
| Inferior occipital gyrus | L | -26 | | | -88 | -4 | | | |
| Middle cingulum | R | 6 | | | -36 | 48 | | | |
| Middle frontal gyrus | R | 32 | | | 26 | 42 | | | |
| Precentral and postcentral gyri | R | 28 | | | -26 | 56 | | | |
| Superior occipital gyrus | R | 22 | | | -84 | 14 | | | |
| Superior occipital gyrus | R | 18 | | | -100 | 18 | | | |
| Superior temporal gyrus | R | 46 | | | -14 | 0 | | | |
|  |  |  | | |  |  | | | |
|  |  |  | | |  |  | | | |
|  |  |  | | | MNI Coordinates | | | |  |
| *Typical Development > Autism, Physical > Social* | Hemi. | X | | | Y | Z | | | |
| Inferior occipital gyrus | L | -28 | | | -86 | -2 | | | |
| Inferior parietal lobule | L | -40 | | | -36 | 40 | | | |
| Inferior temporal gyrus | R | 44 | | | -58 | -6 | | | |
| Middle frontal gyrus | R | 34 | | | 30 | 40 | | | |
| Precentral gyrus | R | 18 | | | -18 | 68 | | | |
| Precentral gyrus | R | 32 | | | -24 | 66 | | | |
| Supplementary motor area | R | 16 | | | -4 | 62 | | | |
|  |  |  | | |  |  | | | |
|  |  |  | | |  |  | | | |
|  |  |  | MNI Coordinates | | | |  | | |
| *Typical Development > Autism, Social > Physical* | Hemi. | X | | | Y | Z | | | |
| Inferior frontal gyrus–orbitalis, triangularis | L | -52 | | | 20 | 2 | | | |
| Middle temporal gyrus |  | 52 | | | -2 | -26 | | | |
| Anterior cingulum |  | 4 | | | 40 | 10 | | | |
| Insula |  | 30 | | | 12 | -18 | | | |
| Hippocampus |  | -28 | | | -14 | -16 | | | |
| Insula |  | -28 | | | 14 | -10 | | | |
| Hippocampus |  | -30 | | | -26 | -10 | | | |
| Superior temporal pole and inferior frontal gyrus |  | -32 | | | 14 | -22 | | | |
| Pallidum |  | -8 | | | 8 | -2 | | | |
| Middle temporal gyrus |  | -58 | | | -14 | -20 | | | |
| Superior temporal pole |  | -46 | | | 8 | -22 | | | |
| Inferior frontal gyrus–orbitalis |  | -32 | | | 34 | -10 | | | |
| Inferior frontal gyrus–triangularis |  | -10 | | | -90 | 30 | | | |
| Precuneus |  | 2 | | | -54 | 22 | | | |
